# Fungi-sensitized individuals have unique profiles where Alt a 1 dominates promoting response to grass, ragweed and cat allergens

**DOI:** 10.3389/falgy.2024.1438393

**Published:** 2024-08-23

**Authors:** Viktoriia Kalyniuk, Victoria Rodinkova, Serhii Yuriev, Vitalii Mokin, Arsen Losenko, Mariia Kryvopustova, Diana Zabolotna, Inna Gogunska

**Affiliations:** ^1^ Department of Allergology, SI Institute of Otolaryngology n.a. Prof.O.S. Kolomiychenko of NAMS of Ukraine, Kyiv, Ukraine; ^2^ Department of Pharmacy, National Pirogov Memorial Medical University, Vinnytsya, Ukraine; ^3^ Department of Allergology, Medical Centre DIVERO, Kyiv, Ukraine; ^4^ Department of System Analysis and Information Technologies, Vinnytsia National Technical University, Vinnytsia, Ukraine; ^5^ Department of Pediatrics No2, Bohomolets National Medical University, Kyiv, Ukraine

**Keywords:** *Alternaria*, fungal sensitization, fungal allergens, polysensitization, allergen clustering, Bayesian network analysis, K-means++ clustering, molecular diagnostics of allergy

## Abstract

**Introduction:**

The aim of our work was to determine comprehensively the sensitization profile of patients hypersensitive to fungal allergenic components in the Ukrainian population, identifying features of their co-sensitization to allergens of other groups and establishing potential relationships between causative allergens and their ability to provoke this hypersensitivity.

**Methods:**

A set of programs was developed using Python and R programming languages, implementing the K-means++ clustering method. Bayesian networks were constructed based on the created clusters, allowing for the assessment of the probabilistic interplay of allergen molecules in the sensitization process of patients.

**Results and discussion:**

It was found that patients sensitive to fungi are polysensitized, with 84.77% of them having unique allergological profiles, comprising from 2 to several dozen allergens from different groups. The immune response to Alt a 1 may act as the primary trigger for sensitization to other allergens and may contribute to a high probability of developing sensitivity to grasses (primarily to Phl p 2), ragweed extract, and the Amb a 1 pectate lyase, as well as to pectate lyase Cry j 1 and cat allergen Fel d 1. Individuals polysensitized to molecular components of fungi were often sensitive to such cross-reactive molecules as lipocalins Fel d 4 and Can f 6, as well. Sensitivity to Ambrosia extract which dominated in the development of sensitization to ragweed pollen indicating the importance of different allergenic components of this plant's pollen. This hypothesis, along with the assumption that Phl p 2 may be the main trigger for sensitivity to grasses in patients with *Alternaria* allergy, requires further clinical investigation.

## 1 Introduction

Allergenic sensitization to fungi is a significant factor in allergic rhinitis and asthma (1). Among the main allergens that cause the first type allergic reaction, house dust mites (2), plant pollen, animal epidermis (3), mold and some food products are considered the most important (4, 5). According to scientific observations, for example, in Spain, molds are the fourth important source of sensitization in allergic respiratory diseases, which is concedant only to plant pollen, dust mites and animal epidermal allergens in terms of the number of sensitive people. Mold allergens can cause IgE-mediated allergic rhinitis (AR), asthma (6, 7), and atopic dermatitis (3). There is also scientific evidence that sensitization to certain fungi, such as *Aspergillus* and *Alternaria* (8) is associated with the severity of asthma attacks (9). *A. alternata* and its main allergen Alt a 1 is considered the most important fungal allergen in the world (10, 11), associated with the development of respiratory allergies and asthma (12, 13).

Researches conducted in various regions of the world (11, 14) have shown that sensitization to mold is quite common, especially in patients with respiratory allergies (15, 16). But the exact indexes of sensitization to mold allergens prevalence is unknown. Figures vary from 3 to 10% of the total population (17, 18). Currently, it has been established that the development of allergies is most often tested by 4 types of molds: *Alternaria*, *Cladosporium*, *Penicillium* notatum and *Aspergillus* (19, 20).

According to many studies, *Alternaria alternata* is one of the most important sensitizing molds in Europe (21). Hypersensitivity to this fungus has also been shown to be associated with an increased risk of asthma (22) and high FeNo levels (23).

Previously, there were no reliable data on the sensitization of the population of Ukraine to the allergens of *Alternaria* fingus spores. In recent years, due to to the latest methods of ALEX molecular allergy diagnosis, Ukrainian scientists have conducted studies that proved that the level of sensitization to this allergen in the country is quite high and amounts up to 23.3% (24). *Alternaria alternata* spores are known as powerful allergens. Atmospheric concentrations of *Alternaria* are high and very high from May to September, with peaks in July, when airborne grass and weed pollen levels are high. Mold-sensitive patients are often polysensitized to several species of fungi and to allergens from other sources (25). It is also known that fungi are able to activate the immune system and increase the inflammatory response caused by other allergens, such as pollen (8). Allergy to molds can take the form of hay fever. Hence, hypodiagnosis of sensitization to mold negatively affects the effectiveness of treatment of such patients (26).

Therefore, the objective of our work was to comprehensively determine the sensitization profile of patients hypersensitive to fungi allergenic components in the Ukrainian population with the determination of the features of their cosensitization to allergens of other groups and the establishment of possible relationships between the causative allergens in their ability to provoke this hypersensitivity.

## 2 Materials and methods

### 2.1 Participants

In order to solve the objective set, we analyzed the data of 3,349 fungi molecular components sensitive people who were tested by ALEX method in 17 regions of Ukraine in 2020-2022. Patients with a medical history of allergic rhinitis, chronic urticaria, allergic dermatitis, asthma were eligible for inclusion in the study. Exclusion criteria encompassed the absence of any allergy-related medical history and a lack of sensitization to fungal allergens (Table 1).

**Table 1:** Concomitant allergic diseases in patients who had sensitization to fungal allergens^a^.

Allergic diseases	Number (%) of children (*n* = 2,607)	Number (%) of adults (*n* = 742)
Bronchial asthma	1,043 (40)	215 (29)
Allergic rhinoconjunctivitis	2,007 (77)	608 (82)
Allergic urticaria/angioedema	235 (9)	82 (11)
Food allergy to vegetable proteins	547 (21)	67 (9)
Food allergy to animal proteins	156 (6)	-
Allergic dermatitis	235 (9)	37 (5)
Drug allergy	-	30 (4)
History of anaphylaxis	2 (0,07)	4 (0,5)

^a^ Several nosological forms could be observed in one patient.

The decision to employ component-resolved molecular allergy diagnostics was determined by the attending physician when presented with symptoms indicative of the aforementioned diseases.

The test results obtained from the ALEX at the Medical Centre, DIVERO, Kiev, Ukraine, were utilized in the study. ALEX (Allergen Explorer) is an array of allergens spotted on a solid phase by the use of nanoparticles. ALEX contains 295 reagents (117 allergen extracts and 178 recombinant or highly purified molecules). Thus, this chip, like the Microtest, contains second-level diagnostics (represented by extract allergens) and third-level diagnostics (represented by single molecules). This microarray allows the measurement of an IgE profile including “whole” allergens and recombinant or purified allergen proteins in a single chip (27).

### 2.2 Ethics

All patients signed informed consent before testing. Among others, it included a paragraph about the possible usage of impersonalized patient data for scientific purposes. The Bioethics and Deontology Committee at the State Institution “Prof. O.S. Kolomiychenko Institute of Otolaryngology of the Academy of Medical Sciences of Ukraine”, approved the study protocol No. 1/22-1 dated by June 3, 2022.

In this study, we did not aim to collect and analyze detailed symptomatology of patients in the context of its correspondence with the molecular test results. Instead, the main attention was paid to the comprehensive analysis of sensitization in fungi-responsive patients in the Ukrainian population and to the interplay of the factors that can influence it.

### 2.3 Variables and data sources

The patients who were sensitive to at least one of the fungal constituents of the ALEX multiplex allergy test were chosen for the subsequent analyses. These allergens were as follows: Alt a 1 (major allergen, class undefined) and Alt a 6 (enolase) of *Alternaria alternata*; Asp f 1 (representative of the mitogillin family), Asp f 3 (peroxisomal protein), Asp f 4 (class undefined), Asp f 6 (Mn superoxide dismutase) of *Aspergillus fumigatus*; Cla h (extract) and Cla h 8 (mannitol dehydrogenase) of *Cladosporium herbarum*; Mala s 5 (class undetermined), Mala s 6 (cyclophilin) and Mala s 11 (Mn superoxide dismutase) of *Malassezia sympodialis*, as well as extracts from *Penicillium chrysogenum* (Pen ch) and *Saccharomyces cerevisiae* (Sac c). The categorization of allergen molecules into classes adheres to The World Health Organization and International Union of Immunological Societies (WHO/IUIS) Allergen Nomenclature database (http://allergen.org, Accessed January 28, 2024). According to the reference values of the ALEX test, the sensitization threshold was determined at 0.31 kU/L.

Data on sensitization of the studied group of patients to other allergenic components available for testing in ALEX were preserved. In total, ALEX allows to determine sensitivity to 295 allergens in 7 groups: pollen, molds & yeasts, mites and cockroaches, dander & epithelia, insect venom, food (cereals & seeds, egg & milk, fruits, vegetables, legumes and nuts, meat, seafood, spices) and others, which, among other ones, include allergens of Baker's yeasts, Hom s lactoferrin (CCD), latex, pigeon tick (*Argas reflexus)* and weeping fig (*Ficus benjamina*).

### 2.4 Data analyses

For solving the problem, a set of programs was developed in the Python and R programming languages, which implement the K-means++ clustering method. After its application, on the basis of the created clusters, Bayesian networks were built, which made it possible to establish the probabilistic interplay of allergen molecules in the process of sensitization of patients.

K-Means clustering is a method of data grouping, where each cluster is characterized by its center of values. The K-means++ method is an improved initialization technique for the K-means clustering algorithm. It allows to choose the initial centers of clusters so that the K-means algorithm increases the speed of finding convergence of values and finds the best ways of clustering so that every value, in contrast with widely used fuzzy clustering, can be placed to one cluster only.

Furthermore, examples of using K-means clustering to characterize patients' allergenic sensitization patterns are already known in the literature (28). However, unlike the mentioned authors, we employed the most advanced and precise version, K-means++.

The K-means++ initialization process involves randomly selecting the first cluster center from a given data set. Next, for each subsequent center of the cluster, a new center is selected from the data set with a probability proportional to the square of the distance from each point to its nearest center. This process is repeated until all K-centers are selected, ensuring their uniform distribution within the data.

The silhouette score metric was used to assess the quality of clusters formed by the K-means++ algorithm. This metric involves measuring of a random cluster similarity point with its own cluster (cohesion) compared to other clusters (distance). A higher silhouette score indicates a higher quality of cluster formation. After calculating the silhouette score for different K values, the K value that gives the highest silhouette score value is considered the optimal number of clusters for the selected data set. This value of K produces well-defined, significant clusters with a high level of clustering quality and a balance between cohesion and remoteness of data points.

When choosing the K value with the highest silhouette score, the optimal number of clusters for further research was obtained.

A Bayesian network was constructed based on the clusters obtained using K-Means++. Each cluster can be considered as a system of this network, and the points in the cluster—as its nodes.

All the steps were done using Kaggle platform (Supplementary S1, https://www.kaggle.com/code/vbmokin/fungi300-research). Below is a summary of the steps in the Kaggle code:

1. Data Sample input.
2. Determination of Optimal number of Clusters using Sihouette method.
3. Execution of Clustering. Includes initial clustering around the cores where the number of cores matches the number of clusters determined by the Silhouette method.
4. Clusters Analysis. It includes clusters evaluation for patterns obtained and check if any clusters are too large.
5. Reclustering. Clusters deemed too large treated as new data samples and they repeat the process starting from step 1. This cycle continues until all clusters are appropriately sized.
6. For clusters which not exceed 12 components, Bayesian modelling can be used.

Bayesian modeling allows taking into account uncertainty and probabilistic relationships within clusters based on probability theory. Hierarchical connections between nodes within the Bayesian network make us possible to draw conclusions about potential factors and ways of developing sensitization of patients both to specific molecules and to a group of allergens.

## 3 Results

### 3.1 Characteristics of patients

As it was mentioned above, there were 3,349 individuals in the studied sample. Children under 18 made up 77.84% of them (2,607 examined), adults—22.16% (742 patients). The number of children in the sample exceeded the number of adults by 3.51 times. Detailed age distribution and sensitization to fungal components in this sample are described in our previous paper (29).

The largest number of patients—2,659 or 79.30% were sensitive to Alt a 1. As well as 2,082 persons or 62.09% of patients sensitive to fungi had sensitization to Alt a 1 as the solitary component of fungi. The remaining 1,267 patients (37.91%) were sensitive to other combinations of fungal molecules and other allergens (Table 2).

**Table 2:** Sensitivity to different fungal components in the studied sample.

Molecular component	Number (%) of patients sensitive to the allergen	Number (%) of patients sensitive to this fungal allergen only
Alt a 1	2,659 (79.30)	2,082 (62.09)
Alt a 6	126 (3.76)	22 (0.66)
Asp f 1	36 (1.07)	14 (0.42)
Asp f 3	228 (6.80)	27 (0.81)
Asp f 4	70 (2.09)	29 (0.86)
Asp f 6	133 (3.97)	10 (0.30)
Cla h	177 (5.28)	15 (0.45)
Cla h 8	343 (10.23)	76 (2.27)
Mala s 5	165 (4.92)	91 (2.71)
Mala s 6	354 (10.56)	134 (4.00)
Mala s 11	187 (5.58)	51 (1.52)
Pen ch	42 (1.25)	6 (0.18)
Sac c	104 (3.10)	16 (0.48)

### 3.2 Analysis of data on the sensitivity of the Ukrainian population to fungal allergens

**Figure 1:**
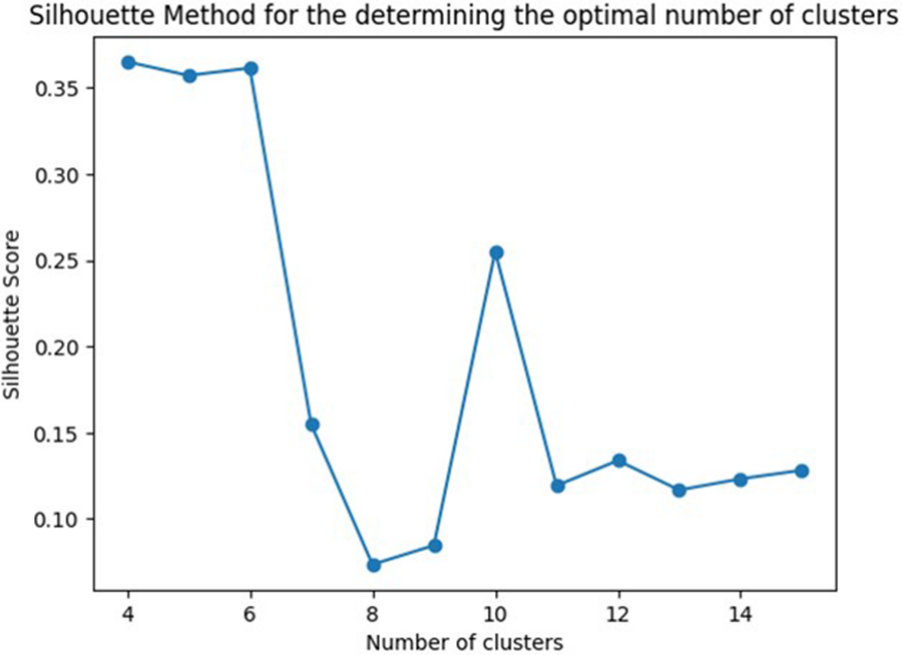
The optimal number of clusters for grouping patients with fungal sensitization, determined using the silhouette method.

Among all 295 allergenic components of the ALEX test, the Sihouette method was used to determine the optimal number of clusters into which the existing database could be grouped basing on the most frequent sensitivity to certain allergenic components. The most optimal number of clusters was 4 (Figure 1).

**Figure 2:**
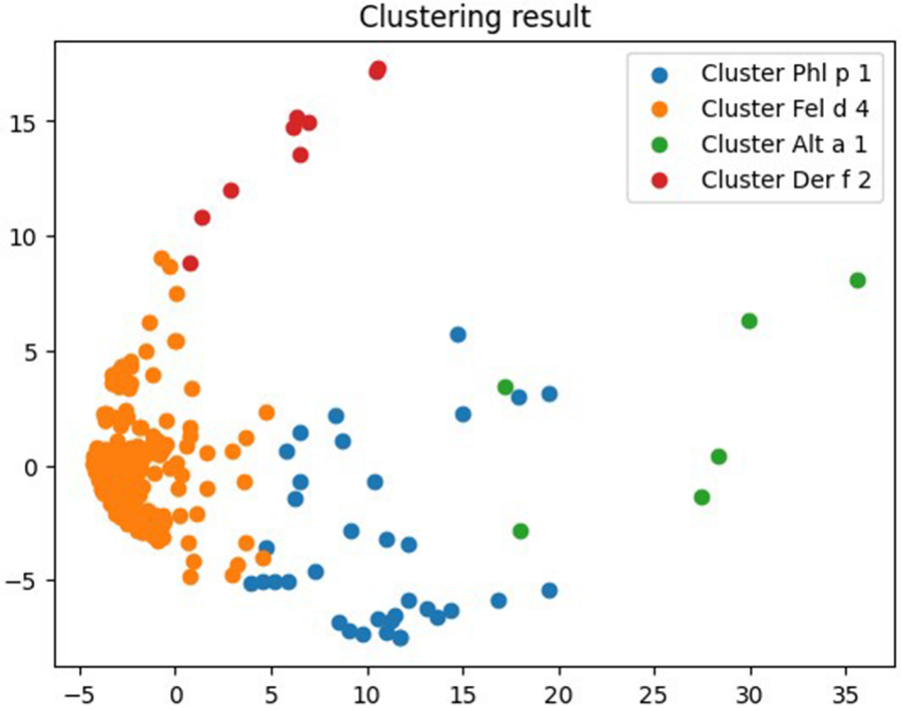
Results of molecular components clustering to which all patients with fungi sensitization were sensitive to.

Thus, clustering of the database was carried out with the formation of 4 clusters. Fungal molecules were included in only two of the four clusters obtained, numbered by the system from 0 to 3. The clusters were designated by the name of the molecular component to which sensitization was observed most often in this cluster (Figure 2, Supplementary S1, https://www.kaggle.com/code/vbmokin/fungi300-research).

In particular, cluster No. 1 included all 12 molecules and fungi extracts available for analysis, except for Alt a 1. Together with them, cluster 1 included 250 or 83.89% of the allergenic components present in the ALEX test.

The molecule to which the most patients were sensitized in this cluster was Fel d 4. It was followed by Can f 6, Cup a 1, Mala s 6 and Cla h 8, respectively.

Alt a 1 was included in cluster No. 2. Along with it, this cluster included ragweed extract Amb a and the main allergen molecules of ragweed Amb a 1, cryptomeria Cry j 1, cat Fel d 1, and timothy Phl p 2.

The most numerous was sensitization to Alt a 1 in it. The second and third places were occupied by Fel d 1 and Amb a 1, respectively.

In the zero cluster, the leading molecule was Phl p 1, in the third—Der f 2, fungal molecules were not observed in both clusters (Table 3).

**Table 3:** ALEX test molecular components to which the most individuals in each cluster were sensitive.

Cluster 0		Cluster 1		Cluster 2		Cluster 3	
Compo-nent	No (%) of sensitized	Compo-nent	No (%) of sensitized	Compo-nent	No (%) of sensitized	Compo-nent	No (%) of sensitized
Phl p 1	1,133 (33.83)	Fel d 4	458 (13.67)	Alt a 1	2,661 (79.45)	Der f 2	845 (25.23)
Lol p 1	1,022 (30.52)	Can f 6	398 (11.88)	Fel d 1	1,816 (54.22)	Der p 2	837 (24.99)
Bet v 1	988 (29.50)	Cup a 1	375 (11.19)	Amb a 1	1,656 (49.44)	Der f 1	663 (19.79)
Cyn d 1	866 (25.86)	Mala s 6	357 (10.65)	Amb a	519 (15.49)	Der p 23	655 (19.55)
Fag s 1	843 (25.17)	Cla h 8	344 (10.27)	Phl p 2	1,151 (34.36)	Der p 1	629 (18.78)
Cyn d	821 (24.51)	All other fungal molecules, 10+		Cry j 1	1,032 (30.81)	Lep d 2	595 (17.76)

Since cluster 1 included a large number of fungal molecules, it, in turn, was also subjected to clustering. The optimal number of clusters here was 14 (Supplementary S1, https://www.kaggle.com/code/vbmokin/fungi300-research). But among them, only 2 clusters were found, which included fungi. Cluster 1 of this secondary clustering included 11 fungal molecular components in addition to Alt a 1, which was separated in the primary cluster, and Mala s 6, which was included in cluster 7 of this second clustering stage.

**Figure 3:**
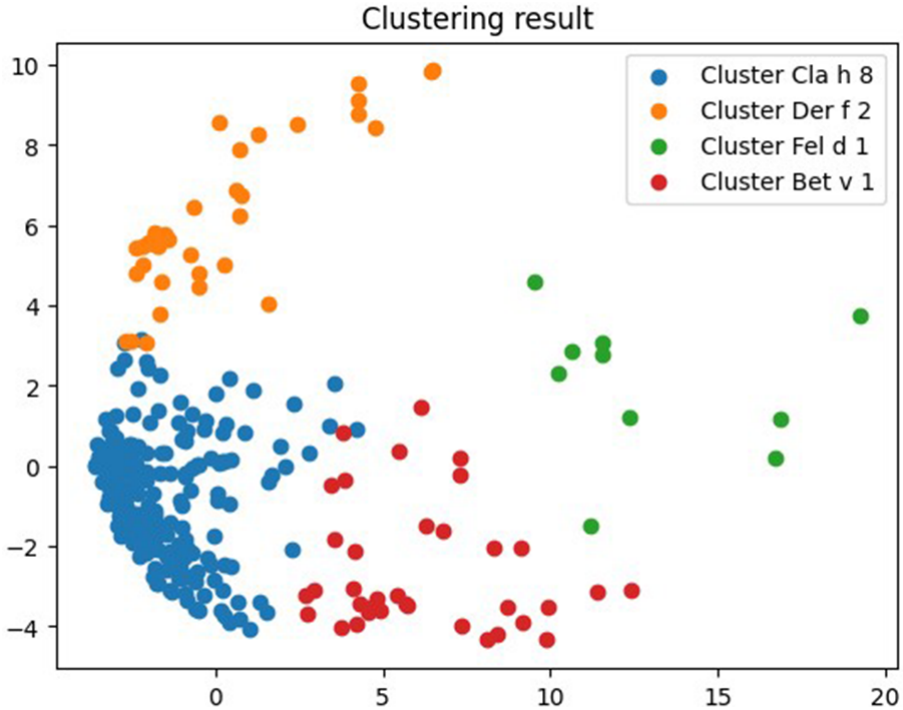
Clustering results for molecular components to which patients with fungal polysensitivity were sensitive.

As the number of allergens in the cluster with the largest number of fungal components exceeded 200 molecules at the initial stages of clustering, and subsequent stages separated fungal molecules into individual clusters, which made it impossible to establish the features of co-sensitization to them, we performed clustering separately for those who was sensititive to various fungal components, but not to Alt a 1 only. According to Table 1, there were 37.91% of such patients. As a result, 4 clusters were obtained (Figure 3, Supplementary S1, https://www.kaggle.com/code/vbmokin/fungi300-research).

But even in this case, the null cluster Cla h 8 was the most numerous and contained 206 allergens, among which there were 9 molecular components of fungi (Alt a 6, Asp f 1, Asp f 3, Asp f 6, Cla h, Cla h 8, Mala s 11, Pen ch, Sac c). Cluster No. 1 Der f 2 contained 36 components, among which two—Asp f 4 and Mala s 5—belonged to fungi. The third cluster Fel d 1 was the least numerous and contained 10 allergens, only one of which—Alt a 1—belonged to fungi. The composition of this cluster for individuals sensitive to various fungal components was similar to the composition of Alt a 1 cluster for the general sample. The last cluster, Bet v 1, contained the fungal molecule Mala s 6 (Figure 3, Supplementary S2).

### 3.3 Analysis of individual profiles of patients sensitized to fungi

**Figure 4:**
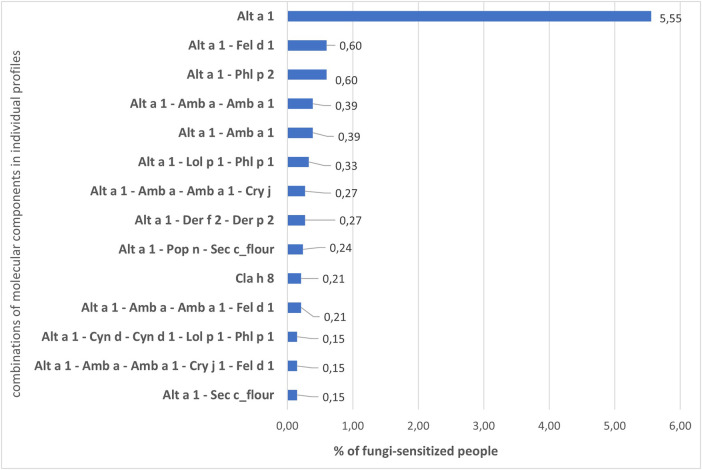
Percentage of patients with fungal sensitization who are sensitive to different combinations of molecular components.

In the individual profiles of patients, as Bayesianin the general sample, sensitivity to Alt a 1 prevailed. The number of people sensitive only to Alt a 1 of 295 ALEX components was 5.55% of the entire fungi-sensitive sample. The share of sensitized to other combinations of allergens was much lower and started from 0.60% (Figure 4), 2,839 patients (84.77%) had their own unique sensitization profile. The profile of each of these patients did not match the profiles of other ones and could include from 2 to several dozen allergens of different groups (Supplementary S3).

### 3.4 Analysis of probabilistic relationships between sensitization to different molecular components of fungi using modeling

We noticed that in the general sample and in the sample of fungi-polysensitized, the Alt a 1 component fell into clusters with a similar set of molecules. In the first case, these were Alt a 1, Amb a, Amb a 1, Cry j 1, Fel d 1, Phl p 2. A similar cluster for polysensitized individuals was somewhat wider and contained, in addition to those listed, molecules of grass of the I group Cyn d 1, Lol p 1, Phl p 1 and Cyn d extract. All these molecules were included in the list of the most frequent combinations of allergens in individual profiles of patients (Figure 4).

For clusters with the mentioned molecules, we performed Bayesian modeling to establish a hierarchical structure of sensitization and a conditional distribution of probabilities of sensitization to some allergens against the background of sensitization to others. In both cases, the Alt a 1 molecule was identified as the root node for Bayesian modeling.

A directed acyclic graph for the total sample showed that sensitization to Alt a 1 was key. It determined the probability of developing sensitivity to Phl p 2, which, in turn, caused sensitization to ragweed extract Amb a and the molecule Amb a 1.

**Figure 5:**
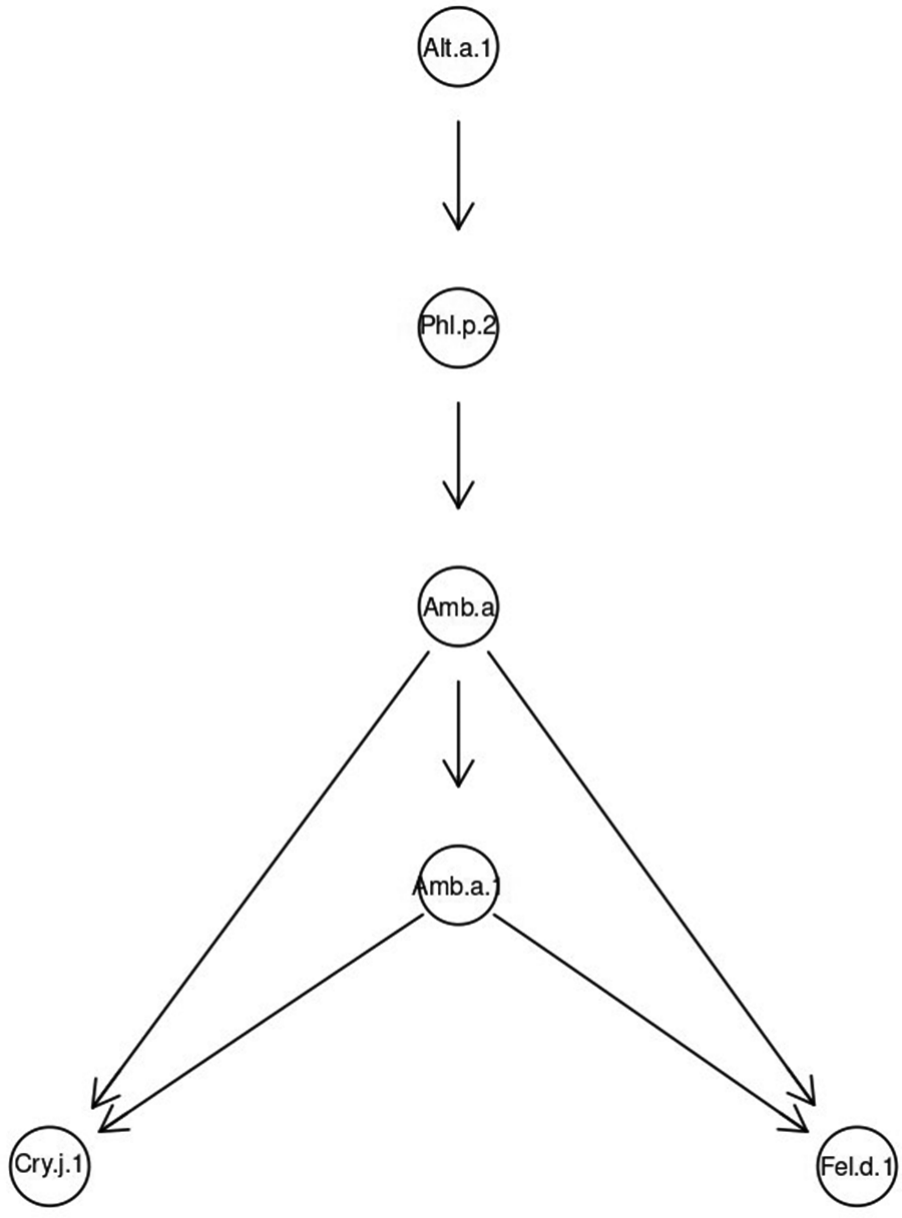
Bayesian directed acyclic graph of probabilistic relationships between allergenic components in a general sample of patients sensitive to fungi.

Sensitization to Cry j 1 and Fel d 1 molecules developed after acquiring sensitivity to Amb a 1 (Figure 5).

**Figure 6:**
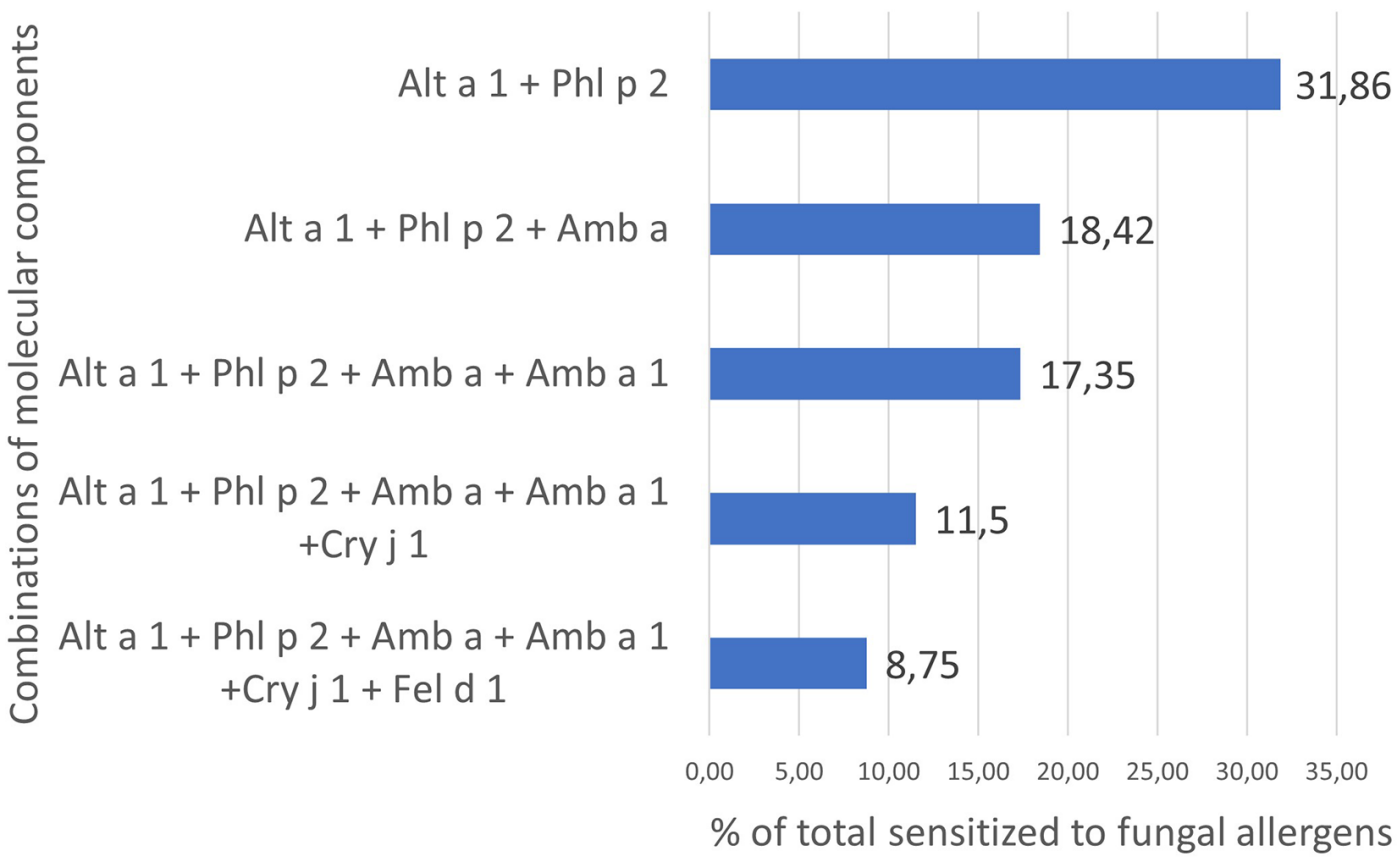
Percentage of fungi-sensitized individuals with molecular components of cluster 2 in their profiles.

According to descriptive statistics, 1,067 or 31.86% of people with sensitivity to fungal components were simultaneously sensitized to Alt a 1 and Phl p 2. Moreover, 83.22% of them were children. The share of simultaneously sensitized to other combinations gradually decreased and amounted to 8.75% for those sensitized to all 6 molecules of cluster 2 of the total sample (Figure 6).

The Bayesian network determined that the probability of sensitization to Alt a 1 was 79.39% in the sample. This matched with real data (79.30%) of sensitization to this allergen almost to hundredths of a percent (Table 3). The calculated probability of simultaneous sensitivity to Alt a 1 and Phl p 2 was also similar to the real one (Figure 6) and amounted to 40.12%. Sensitivity to Amb a depended on sensitization to Phl p 2. But the highest probability (CPD = 61.27%) was the simultaneous absence of sensitization to both of these components. The probability of simultaneous sensitivity to the extract and the major ragweed allergen was 94.25%.

On the other hand, in the presence of sensitization to the extract and the main ragweed allergen Amb a 1, the probability of sensitivity to Cry j 1 was 62.86%. Moreover, if there was no sensitivity to Cry j 1, then the probability of simultaneous sensitivity to the main ragweed allergen and its extract was only 37.13%. Against the background of the presence of sensitivity to both Amb a 1 and Amb a, the probability of sensitivity to the main cat allergen Fel d 1 was 71.88%. If sensitivity to only ragweed extract was present, the probability of sensitivity to Fel d 1 was 75.79% (Supplementary S4, version 2 https://www.kaggle.com/code/vbmokin/bnlearn-on-r-for-fungi300).

**Figure 7:**
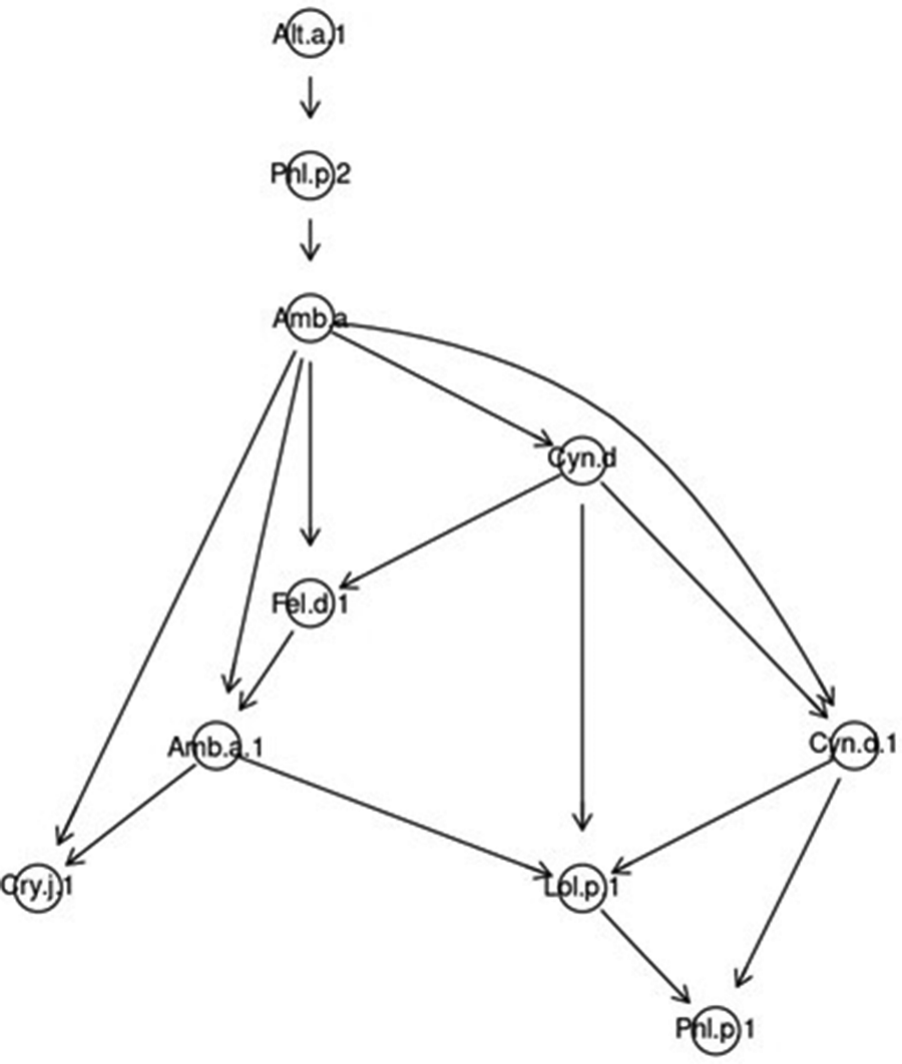
Bayesian directed acyclic graph of probabilistic relationships between allergenic components in a sample of patients polysensitized to fungi.

In the sample of patients polysensitized to fungal molecules, the patterns were similar. The leading role was played by Alt a 1 and Phl p 2, which determined sensitization to ragweed extract Amb a. Molecules Amb a 1, Fel d 1 and Cyn d formed nodes of higher level. In particular, *Cynodon* extract caused sensitivity to all molecules of group I grasses and to the main cat allergen. Amb a 1 dominated over Cry j 1 and Lol p 1, which, in turn, determined sensitivity to Phl p 1 (Figure 7).

Alt a 1 regulated sensitivity to Phl p 2 in such a way that the probability of simultaneous sensitization to these two molecules in polysensitized people was 74.33%. The probability of simultaneous sensitivity to Phl p 2 and Amb a extract was 62.21%. In the presence of sensitivity to Amb a 1, the probability of sensitivity to Cry j 1 was 35.17%, and in the presence of sensitivity to both the main ragweed allergen and its extract, this probability increased to 64.83%. The probability of simultaneous sensitivity to Amb a and Amb a 1 against the background of both the presence and absence of sensitivity to Fel d 1 were approximately the same and amounted to about 93.00%.

In the absence of sensitivity to *Cynodon* extract and Cyn d 1, the probability of sensitivity to *Ambrosia* extract was 94.48%. If a person was sensitive to Cyn d, he had an 86.28% probability of being simultaneously sensitive to Fel d 1 and Amb a. In the absence of sensitivity to the main molecule and *Cynodon* extract, the probability of the absence of simultaneous sensitization to Amb a 1 and Lol p 1 was 91.02%. And against the background of sensitivity to Amb a 1, the probability of sensitization to Lol p 1 was up to 81.83%. In the presence of sensitivity to Cyn d and Cyn d 1, the probability of simultaneous sensitization to Amb a 1 and Lol p 1 was 88.65%. And almost 100% (99.96%) was the probability of sensitization to Phl p 1 in the presence of patient sensitivity to Lol p 1 and Cyn d 1 (Supplementary S4, version 4 https://www.kaggle.com/code/vbmokin/bnlearn-on-r-for-fungi300).

## 4 Discussion

To authors knowledge, the presented study is the first detailed investigation of the nature of complex multicomponent sensitization in patients sensitive to fungi, which is an undeniable strength of the presented work. Other strong points are the simultaneous clustering and Bayesian modeling to establish a hierarchical relationship between molecular components that could indicate the sequence of development of sensitization to them in patients with fungal sensitivity.

In the current study, we did not conduct a detailed comparison of the sensitization of patients with the nature of their symptoms, which can in some cases be considered as a limitation of this research. However, such a comparison was not the goal of our work, and the analysis of detailed symptoms would significantly complicate the perception of the result. However, even our general data on patient symptoms correlate well with the results of clustering. After all, people sensitized to fungi experienced respiratory symptoms most frequently (Table 1). These symptoms could be caused by molecules from the Fel d 4 and Alt a 1 clusters which, in addition to fungal allergens, included exclusively respiratory allergens (Table 3). Moreover, the Fel d 4 cluster, which included all fungal molecules except Alt a 1, was quite dense (Figure 2). This may indicate that its components often act together, which confirms our results that patients with sensitivity to fungi are polysensitized. Such polysensitization can be associated with cross-reactive molecules of lipocalins Fel d 4 and Can f 6 that were also included in the Fel d 4 cluster which contained all allergenic components of fungi, except for Alt a 1.

The fact of polysensitization of patients in the sample described by us correlates with the studies of other authors, who proved that sensitization to *Alternaria alternata* is usually associated with sensitivity to other allergens (30). We found that polysensitization of patients with sensitivity to fungi can have a unique individual character, although it is characterized by certain regularities. In particular, our studies confirm the conclusions of other authors that *Alternaria alternata* and its main allergen Alt a 1 are the most important among fungi allergens in allergic diseases (8, 31).

It is the leading role of Alt a 1 that we discovered, to which solely two thirds of fungi-sensitive patients were sensitized, may explain why Alt a 1 was assigned to one cluster, and all other fungal allergens to another one.

Alt a 1 itself fell into the same cluster with ragweed and grass allergens. The dominant position of Alt a 1 in this cluster, determined by the Bayesian network, correlates with the data of Kim HK et al., who described the ability of fungi to activate the immune system and increase the inflammatory response caused by other allergens, such as grass pollen (25).

In our study, it seems that Alt a 1 is the allergen that causes further sensitivity to grass, namely to Phl p 2—the genuine allergen of group II grasses (32). The ability of Alt a 1 to trigger immune response correspond with the clinical data of other authors (30, 33). They described both ability of Alt a 1 to trigger immune response (8, 25) and to provoke grass allergy (33).

Phl p 2 is a primary elicitor of allergy to grass pollen and can provoke the development of sensitization to them independently of other allergens according to our own studies of the Ukrainian population (32).

Calculated by us high number of patients sensitive simultaneously to Alt a 1 and Phl p 2, with almost 90% children among them, confirms the hypothesis of the leading role of the *Alternaria* allergen in the ability to initiate the response of the immune system (33), especially—during chilhood.

Sensitization to *Alternaria* can be associated, first of all, with sensitivity to allergens of grasses and ragweed specifically because the highest concentration of *Alternaria* spores is observed in the term from June to October, which coincides with the florescence period of grasses and weeds (ragweed and mugwort), in particular in Ukraine (34).

It is notable that allergens Alt a 1, Amb a 1 and allergens of grasses from groups I and II were part of the same cluster in the general sample and in the sample of patients polysensitized to fungi.

Moreover, the hierarchical sequence of the sensitization occurrence, determined by the Bayesian network, corresponds to the sequence of florescence of plants—first Poaceae, then *Ambrosia*.

The trigger for such polysensitization can be fungi which occupied the highest level in the hierarchy of allergy triggers in our Bayesian modeling, where Alt a 1 was the paternal node for each of the analyzed acyclic graphs—for the entire sample and for patients polysensitized to various molecular components of fungs.

The correctness of the proposed hypotheses can be confirmed by the general correctness of the performed simulation. In particular, the probability of the occurrence of sensitivity to Alt a 1, calculated by the Bayesian network, practically coincided with the actual sensitivity of patients to this allergen. It is also worth noting that the system combines into one cluster the allergens Amb a 1 and Cry j 1, which belong to the class of pectate lyases, as well as the extract of Amb a, which includes the leading allergenic component Amb a 1. Moreover, sensitivity to the extract is higher in the hierarchy of the Bayesian network than sensitivity to the major allergen Amb a 1. That is, not only sensitivity to Amb a 1, but also to other allergenic components of the ragweed pollen is important in the development of sensitization to this plant (35).

This was also confirmed by the levels of probability of sensitization to various allergens against the background of sensitivity to ragweed extract, determined by Bayesian modeling. In general, the probability of sensitivity to Fel d 1, Cry j 1 was higher in the presence of sensitization to ragweed extract, and the risk of sensitivity to *Cynodon* extract was correlated with a higher risk of sensitivity to ragweed extract and Fel d 1. It is worth mentioning that almost 100%-probability sensitization to Phl p 1, in the presence of the patient's sensitivity to Lol p 1 and Cyn d 1 was calculated by the system, which may indicate a confirmation of the clinically known cross-reactivity between allergens of the I group of grasses (36).

The correctness of the conducted clustering is confirmed, in particular, by the fact that cluster 3 of the general sample included all mite allergens, which are an important component of sensitivity in the Ukrainian population and are characterized by high cross-reactivity (37, 38).

Also, according to the literature, there is a homologous similarity between the structure of the main cat allergen Fel d 1 and the *Alternaria* allergen Alt a 1, which the Bayesian network also attributed to one cluster. They are described as allergenic proteins that bind calcium and are capable of oligomerization due to protein-protein interactions, which plays an undeniable role in allergenicity and stability of allergens (39).

In this study, we did not consider cross-reactivity between the fungal allergens. Our aim was to identify the most common combinations of allergens in patients’ profiles (using clustering) and to analyze the possible interrelations of these allergens in their potential to provoke an immune response (using Bayesian analyses). Our findings from both clustering and descriptive statistics show that Alt a 1 typically appears independently in most patients' profiles.

The placement of other fungal molecules in the same cluster can be attributed either to cross-reactivity or to unknown properties of the studied fungal components that may trigger a similar immune response, causing individuals sensitive to one fungus to develop sensitivity to other fungal components as well.

Among the fungal molecules tested, only Asp f 6 and Mala s 11 belong to the same class of Mn superoxide dismutases. The class for Alt a 1, Asp f 4, and Mala s 5 is unknown. Alt a 6 is an enolase, Asp f 1 is mitogilin, Asp f 3 is a peroxisomal protein, Cla h 8 is a mannitol dehydrogenase, and Mala s 6 is a cyclophilin. Cla h, Pen ch, and Sac c are extracts that may contain components cross-reactive with other molecules. For example, enolase Cla h 6 is known among *Cladosporium* allergens, but the specific allergenic molecular components of *Saccharomyces* are still unknown (40). So, cross-reactivity of most of these allergens remains uncertain and was not widely observed in the current study.

Thus, our article demonstrates the most likely combinations of co-sensitization of fungi sensitive people to allergens of different groups. It is demonstrated that individuals who are sensitive to the main allergen of *Alternaria* Alt a 1 are also sensitized to timothy pollen Phl p 2, *Ambrosia* pectate lyases Amb a 1 and Cry j 1, cat allergen Fel d 1. That is, common sensitization to various molecules has been detected, although they belong to different groups and sources of allergens. It is possible that Alt a 1 is able to activate the immune system, condition the development of sensitization and increase the inflammatory reaction caused by other allergens.

Therefore, in patients with sensitization to fungi, in particular to the main allergen *Alternaria* Alt a 1, it is advisable to use multicomponent molecular allergy diagnostics to determine the individual characteristics of sensitization to allergens of other groups, because most of these patients have a unique profile of polysensitization.

## 5 Conclusions

Patients sensitive to fungi are polysensitized and 84.77% of them have unique allergic profiles, which include from 2 to several dozen allergens of different groups.

The immune reaction to Alt a 1 can be the primary trigger for the development of sensitization to other allergens and can cause a high probability of sensitivity to grasses (Phl p 2), to ragweed extract and the major component of Amb a 1 (pectate lyase), as well as to pectate Cry j 1 lyase and Fel d 1 allergen.

In persons polysensitized to molecular components of fungi, this polysensitization can be associated with sensitivity to cross-reactive molecules of lipocalins Fel d 4 and Can f 6.

The development of sensitization to *Alternaria*, grasses, and ragweed in a single patient may be explained by the timing overlap, as the *Alternaria* season begins earlier and continues through the flowering seasons of grasses and ragweed.

In the case of ragweed sensitization, sensitivity to Amb a 1, as well as other allergenic components of the plant's pollen, plays an important role. This hypothesis, along with the suggestion that Phl p 2 may be the main trigger for grass sensitivity in *Alternaria*-allergic patients, requires further clinical study.

*Ambrosia* and *Crypromeria* should be suppressed in Ukraine, by agricultural technologies and stopping further plantation, respectively, due to their high allergenicity in the population.

## Data Availability

The original contributions presented in the study are included in the article/Supplementary Material, further inquiries can be directed to the corresponding author.
